# Supplementary material for: A Likelihood Approach for Real-Time Calibration of Stochastic Compartmental Epidemic Models
Source: PLoS Comput Biol. 2017 Jan 17;13(1):e1005257. doi: 10.1371/journal.pcbi.1005257 (PMC5240920; doi:10.1371/journal.pcbi.1005257)
Supplement: S1 File — (TAR.GZ) [file pcbi.1005257.s014.tar.gz › HSPH_Online-SI-Revision/Additions/IPoi10/n100-extreme_noisy/n100-extreme_noisy_BENCH2_table4.pdf]

1	newly 3 weeks spec	2278.3	2638.2	{1168., 4751.}	45.8631	0.175366	newly 3 weeks cumul	9885.83	11228.5	{5196., 19584.}	42.5236	0.166117	newly 5 weeks spec	1200.81	1341.4	{587., 2463.}	46.105	0.181582	newly 5 weeks cumul	12674.4	14473.7	{6615., 25592.}	43.5862	0.16912	newly 50 weeks cumul	18298.5	17666.7	{7986., 31316.}	40.3132	0.184715
2	newly 3 weeks spec	2435.	1884.59	{801., 3266.}	53.4038	0.280461	newly 3 weeks cumul	9225.	7102.93	{3198., 12717.}	47.6463	0.255804	newly 5 weeks spec	1337.	1207.21	{480., 1880.}	60.3216	0.271653	newly 5 weeks cumul	12421.	9828.68	{4283., 17174.}	50.4037	0.262289	newly 46 weeks cumul	16331.	13915.7	{5657., 23230.}	54.7734	0.263409
3	newly 3 weeks spec	1444.	2202.28	{1012., 4864.}	68.6188	0.192986	newly 3 weeks cumul	6755.	9551.83	{4615., 20329.}	59.7764	0.180788	newly 5 weeks spec	609.	1082.58	{470., 2416.}	87.3825	0.220679	newly 5 weeks cumul	8307.	12185.1	{5820., 26226.}	63.2885	0.184703	newly 51 weeks cumul	9551.	14615.1	{6906., 31925.}	67.7977	0.190669
4	newly 3 weeks spec	766.	1638.58	{876., 2942.}	114.606	0.281515	newly 3 weeks cumul	4576.	8650.21	{4707., 15560.}	90.5965	0.236714	newly 5 weeks spec	254.	583.561	{307., 1069.}	130.225	0.310736	newly 5 weeks cumul	5299.	10210.5	{5522., 18445.}	94.1598	0.243532	newly 55 weeks cumul	5634.	11073.7	{5971., 20118.}	97.8884	0.251124
5	newly 3 weeks spec	1978.	2459.9	{1261., 3987.}	44.9915	0.161938	newly 3 weeks cumul	9313.	10697.8	{5724., 17131.}	39.3801	0.153135	newly 5 weeks spec	894.	1204.07	{597., 1955.}	51.3153	0.171018	newly 5 weeks cumul	11542.	13632.9	{7126., 21916.}	41.2308	0.155843	newly 52 weeks cumul	13327.	16313.5	{8444., 26229.}	43.6284	0.159404
6	newly 3 weeks spec	2370.	1886.3	{1114., 2966.}	39.6998	0.203163	newly 3 weeks cumul	9890.	7938.3	{4761., 12332.}	37.8558	0.193226	newly 5 weeks spec	1132.	1001.18	{577., 1559.}	38.8519	0.181966	newly 5 weeks cumul	12727.	10319.1	{6128., 16021.}	38.1833	0.193121	newly 50 weeks cumul	15600.	12878.6	{7622., 20024.}	38.4295	0.19122
7	newly 3 weeks spec	1951.	3364.73	{1759., 5442.}	76.5521	0.209603	newly 3 weeks cumul	8547.	13014.8	{7162., 20943.}	58.7543	0.177998	newly 5 weeks spec	968.	2001.71	{981., 3212.}	108.484	0.259182	newly 5 weeks cumul	10776.	17639.8	{9442., 28360.}	68.6904	0.196551	newly 48 weeks cumul	12929.	23654.5	{12205., 38086.}	85.749	0.226434
8	newly 3 weeks spec	2287.	2648.19	{1356., 4610.}	43.1031	0.155497	newly 3 weeks cumul	9859.	10750.2	{5907., 18514.}	37.4633	0.147234	newly 5 weeks spec	1178.	1441.99	{728., 2431.}	49.1142	0.162837	newly 5 weeks cumul	12762.	14160.9	{7654., 24568.}	39.5349	0.151035	newly 50 weeks cumul	15717.	17951.4	{9509., 30963.}	42.0712	0.153979
9	newly 3 weeks spec	2292.	3657.39	{1829., 6772.}	66.98	0.196107	newly 3 weeks cumul	10287.	14693.2	{7727., 26029.}	52.5613	0.167589	newly 5 weeks spec	1160.	1988.65	{932., 3910.}	78.5253	0.216969	newly 5 weeks cumul	13098.	19398.4	{9974., 35114.}	57.1947	0.176816	newly 50 weeks cumul	15554.	24559.9	{12318., 45084.}	65.4866	0.193336
10	newly 3 weeks spec	296.	1258.47	{562., 2210.}	325.159	0.548637	newly 3 weeks cumul	1966.	6666.01	{3082., 11377.}	239.065	0.456175	newly 5 weeks spec	92.	449.8	{196., 794.}	388.913	0.60462	newly 5 weeks cumul	2237.	7864.79	{3595., 13456.}	251.577	0.47048	newly 54 weeks cumul	2337.	8537.64	{3878., 14687.}	265.325	0.486507
11	newly 3 weeks spec	2110.	2315.	{1307., 3733.}	35.4059	0.138246	newly 3 weeks cumul	9055.	9351.56	{5293., 15082.}	32.7927	0.136699	newly 5 weeks spec	1137.	1309.63	{674., 2217.}	38.2153	0.141686	newly 5 weeks cumul	11827.	12411.2	{6885., 20286.}	33.6721	0.137882	newly 48 weeks cumul	14734.	16182.1	{8894., 26866.}	35.3147	0.13769
12	newly 3 weeks spec	2018.	3754.22	{1858., 7028.}	90.3212	0.235644	newly 3 weeks cumul	9115.	14623.5	{7740., 26056.}	67.2068	0.196279	newly 5 weeks spec	959.	2128.81	{946., 4133.}	124.246	0.289306	newly 5 weeks cumul	11517.	19604.8	{10034., 35539.}	76.0848	0.212338	newly 49 weeks cumul	13641.	25439.7	{12680., 46968.}	90.4514	0.236916
13	newly 3 weeks spec	3118.	2268.45	{1230., 3858.}	39.055	0.217948	newly 3 weeks cumul	12156.	8832.39	{5009., 14940.}	38.2274	0.212972	newly 5 weeks spec	2008.	1421.61	{712., 2407.}	46.966	0.260987	newly 5 weeks cumul	16586.	12044.1	{6623., 20177.}	38.3798	0.214651	newly 47 weeks cumul	22880.	17296.7	{8780., 28239.}	44.6597	0.235596
14	newly 3 weeks spec	249.	3114.45	{1400., 5377.}	1150.78	1.03016	newly 3 weeks cumul	1934.	13629.9	{6661., 22926.}	604.752	0.79365	newly 5 weeks spec	37.	1480.81	{655., 2554.}	3902.19	1.52658	newly 5 weeks cumul	2082.	17267.5	{8305., 29147.}	729.37	0.860481	newly 52 weeks cumul	2131.	20290.	{9714., 34240.}	852.134	0.917286
15	newly 3 weeks spec	658.	2745.72	{1222., 4486.}	317.283	0.54381	newly 3 weeks cumul	3705.	11405.5	{5703., 19545.}	207.842	0.440219	newly 5 weeks spec	197.	1465.1	{531., 2096.}	643.705	0.732343	newly 5 weeks cumul	4246.	14894.4	{7021., 24755.}	250.786	0.483761	newly 52 weeks cumul	4525.	18226.6	{8120., 28858.}	302.797	0.527124
16	newly 3 weeks spec	902.	2065.79	{943., 3026.}	130.491	0.27213	newly 3 weeks cumul	5020.	9012.67	{4318., 13753.}	85.4683	0.209055	newly 5 weeks spec	342.	981.349	{429., 1363.}	187.074	0.348454	newly 5 weeks cumul	5877.	11435.6	{5379., 17250.}	98.4448	0.227433	newly 52 weeks cumul	6347.	13447.3	{6215., 20035.}	114.288	0.251868
17	newly 3 weeks spec	2633.	4522.81	{2190., 7531.}	78.8239	0.222865	newly 3 weeks cumul	10424.	16453.1	{8903., 26056.}	64.7034	0.198736	newly 5 weeks spec	1461.	2888.3	{1318., 4881.}	103.557	0.259397	newly 5 weeks cumul	13884.	22996.1	{11903., 37459.}	72.3573	0.212384	newly 47 weeks cumul	18108.	32537.	{15998., 53656.}	85.7739	0.234463
18	newly 3 weeks spec	1965.	4323.22	{2263., 7292.}	120.618	0.299818	newly 3 weeks cumul	8760.	16102.2	{9188., 26319.}	85.5194	0.23952	newly 5 weeks spec	847.	2659.02	{1295., 4637.}	213.934	0.434946	newly 5 weeks cumul	10903.	22175.1	{12170., 36992.}	104.375	0.274209	newly 48 weeks cumul	12657.	30446.	{15899., 51358.}	140.741	0.335448
19	newly 3 weeks spec	2260.	4075.85	{1966., 6860.}	85.3387	0.23321	newly 3 weeks cumul	9867.	16027.1	{8387., 26013.}	68.1756	0.203527	newly 5 weeks spec	1026.	2279.98	{1072., 3970.}	123.574	0.292692	newly 5 weeks cumul	12362.	21387.9	{10899., 35357.}	77.7879	0.221068	newly 50 weeks cumul	14680.	27425.4	{13535., 45700.}	90.2669	0.24228
20	newly 3 weeks spec	351.	1729.46	{942., 2627.}	392.724	0.649022	newly 3 weeks cumul	2657.	9177.43	{4860., 14213.}	245.406	0.498194	newly 5 weeks spec	80.	621.955	{326., 989.}	677.444	0.844107	newly 5 weeks cumul	2903.	10843.	{5710., 16573.}	273.509	0.531374	newly 55 weeks cumul	3010.	11756.7	{6204., 17858.}	290.59	0.550309
21	newly 3 weeks spec	1266.	2869.61	{1257., 4404.}	129.161	0.319903	newly 3 weeks cumul	6452.	12840.3	{5957., 19293.}	103.283	0.277845	newly 5 weeks spec	504.	1332.17	{584., 2058.}	165.467	0.37426	newly 5 weeks cumul	7741.	16135.6	{7348., 24385.}	111.976	0.292324	newly 52 weeks cumul	8578.	18848.3	{8467., 28392.}	122.495	0.309943
22	newly 3 weeks spec	1097.	2628.48	{1294., 3836.}	140.437	0.30075	newly 3 weeks cumul	5662.	11576.2	{6303., 17781.}	106.127	0.256822	newly 5 weeks spec	414.	1217.66	{554., 1714.}	194.239	0.368573	newly 5 weeks cumul	6768.	14607.9	{7695., 22112.}	117.224	0.272338	newly 53 weeks cumul	7400.	17017.4	{8771., 25494.}	130.93	0.292822
23	newly 3 weeks spec	2274.	2932.09	{1486., 5130.}	48.6251	0.159499	newly 3 weeks cumul	9195.	11078.4	{5940., 19058.}	41.369	0.14825	newly 5 weeks spec	1192.	1831.71	{863., 3235.}	67.3978	0.18433	newly 5 weeks cumul	12094.	15247.4	{7952., 26241.}	45.6618	0.154643	newly 46 weeks cumul	15344.	21302.6	{10722., 36818.}	54.9074	0.167482
24	newly 3 weeks spec	2818.	2998.41	{1751., 4866.}	38.4814	0.155608	newly 3 weeks cumul	11319.	11333.2	{6899., 17910.}	34.4336	0.151847	newly 5 weeks spec	1477.	1895.14	{1014., 3502.}	50.3058	0.167559	newly 5 weeks cumul	14831.	15614.6	{9281., 24638.}	35.9713	0.14765	newly 46 weeks cumul	19249.	22911.9	{12531., 40027.}	45.3984	0.159485
25	newly 3 weeks spec	1110.	3254.97	{1617., 4570.}	193.24	0.419575	newly 3 weeks cumul	5663.	13044.	{7218., 18425.}	130.682	0.326157	newly 5 weeks spec	432.	1807.92	{872., 2564.}	318.5	0.564567	newly 5 weeks cumul	6746.	17302.8	{9357., 24275.}	156.627	0.368049	newly 49 weeks cumul	7332.	22168.	{11702., 31221.}	202.347	0.434685
26	newly 3 weeks spec	2919.	2595.14	{1467., 3595.}	27.5025	0.13532	newly 3 weeks cumul	11387.	10239.5	{6114., 14163.}	26.3054	0.128076	newly 5 weeks spec	1758.	1527.27	{884., 2213.}	28.9098	0.144541	newly 5 weeks cumul	15461.	13759.7	{8223., 19127.}	26.8853	0.131938	newly 47 weeks cumul	21236.	18535.8	{10812., 25941.}	27.8932	0.139006
27	newly 3 weeks spec	2411.	1338.49	{725., 2367.}	50.1599	0.324933	newly 3 weeks cumul	9503.	5600.49	{3068., 9492.}	47.4462	0.298176	newly 5 weeks spec	1418.	714.559	{387., 1247.}	53.8369	0.362911	newly 5 weeks cumul	12786.	7283.29	{3974., 12344.}	48.876	0.311811	newly 48 weeks cumul	17179.	9183.88	{5023., 15663.}	51.2167	0.336283
28	newly 3 weeks spec	2066.	1890.11	{886., 3276.}	41.4225	0.196597	newly 3 weeks cumul	9289.	8604.09	{4236., 14952.}	39.6817	0.187061	newly 5 weeks spec	1123.	867.158	{395., 1510.}	43.9409	0.238653	newly 5 weeks cumul	12043.	10761.1	{5232., 18719.}	40.0334	0.194765	newly 52 weeks cumul	14773.	12513.4	{5988., 21638.}	40.7393	0.207359
29	newly 3 weeks spec	2511.	3452.71	{1939., 6091.}	49.1836	0.156506	newly 3 weeks cumul	10166.	12921.9	{7717., 21945.}	39.9706	0.138173	newly 5 weeks spec	1397.	2168.23	{1068., 3836.}	64.1387	0.179795	newly 5 weeks cumul	13376.	17838.7	{10225., 30613.}	45.0526	0.148077	newly 46 weeks cumul	17318.	25094.2	{13744., 43547.}	54.5162	0.164568
30	newly 3 weeks spec	1594.	1225.12	{462., 1861.}	55.5244	0.295194	newly 3 weeks cumul	7832.	6353.06	{2536., 9658.}	50.743	0.262117	newly 5 weeks spec	651.	444.597	{154., 667.}	59.0527	0.342757	newly 5 weeks cumul	9505.	7537.25	{2960., 11421.}	51.6575	0.271012	newly 55 weeks cumul	10576.	8196.57	{3211., 12351.}	52.3918	0.279468
31	newly 3 weeks spec	771.	1991.86	{883., 3904.}	160.007	0.325518	newly 3 weeks cumul	4574.	10613.	{4919., 20516.}	134.242	0.301213	newly 5 weeks spec	189.	678.932	{273., 1276.}	259.223	0.446954	newly 5 weeks cumul	5139.	12466.1	{5678., 23946.}	144.268	0.314135	newly 56 weeks cumul	5392.	13388.1	{6082., 25600.}	149.792	0.321465
32	newly 3 weeks spec	858.	2321.77	{1296., 3466.}	170.602	0.399133	newly 3 weeks cumul	4649.	10750.4	{6167., 16069.}	131.242	0.333823	newly 5 weeks spec	288.	1035.51	{590., 1597.}	259.551	0.520379	newly 5 weeks cumul	5438.	13342.6	{7647., 20062.}	145.359	0.358884	newly 53 weeks cumul	5832.	15344.	{8741., 23140.}	163.099	0.388445
33	newly 3 weeks spec	3068.	2670.66	{1416., 4247.}	40.492	0.200972	newly 3 weeks cumul	12287.	11323.9	{6166., 17473.}	38.2082	0.180823	newly 5 weeks spec	1670.	1359.67	{677., 2192.}	42.7447	0.223896	newly 5 weeks cumul	16122.	14600.6	{7843., 22701.}	39.0419	0.187492	newly 51 weeks cumul	20713.	17834.5	{9387., 28026.}	39.7576	0.199569
34	newly 3 weeks spec	2614.	2899.49	{1653., 5252.}	37.7294	0.14442	newly 3 weeks cumul	10179.	11098.9	{6481., 19475.}	33.9681	0.133381	newly 5 weeks spec	1528.	1757.71	{954., 3309.}	41.5927	0.152565	newly 5 weeks cumul	13712.	15143.	{8714., 26831.}	35.5661	0.137224	newly 46 weeks cumul	18212.	20843.	{11794., 37620.}	38.3577	0.141803
35	newly 3 weeks spec	2810.	2495.25	{1273., 3753.}	38.8837	0.188152	newly 3 weeks cumul	11877.	10593.4	{5472., 15943.}	36.107	0.175742	newly 5 weeks spec	1428.	1273.52	{605., 1894.}	40.9104	0.197417	newly 5 weeks cumul	15254.	13658.7	{7016., 20543.}	37.0197	0.179028	newly 51 weeks cumul	18586.	16677.9	{8450., 25096.}	37.8079	0.18206
36	newly 3 weeks spec	2377.	1184.49	{595., 1942.}	56.8855	0.385167	newly 3 weeks cumul	9678.	5021.48	{2675., 8136.}	54.9767	0.36461	newly 5 weeks spec	1295.	631.092	{318., 1029.}	58.4188	0.400704	newly 5 weeks cumul	12751.	6517.46	{3343., 10536.}	55.7695	0.37251	newly 49 weeks cumul	16300.	8132.52	{4182., 13163.}	56.9272	0.384582
37	newly 3 weeks spec	918.	3037.78	{1279., 5945.}	231.384	0.429918	newly 3 weeks cumul	5030.	13530.	{6160., 26556.}	169.603	0.367213	newly 5 weeks spec	306.	1384.72	{517., 2625.}	352.523	0.533268	newly 5 weeks cumul	5864.	16990.2	{7448., 33163.}	190.264	0.390665	newly 54 weeks cumul	6382.	19558.5	{8372., 37725.}	206.921	0.407939
38	newly 3 weeks spec	286.	2421.33	{1255., 3362.}	746.618	0.872731	newly 3 weeks cumul	2079.	11053.7	{6212., 16363.}	431.683	0.690703	newly 5 weeks spec	76.	1191.25	{539., 1487.}	1467.43	1.0804	newly 5 weeks cumul	2297.	13939.7	{7559., 19907.}	506.864	0.73858	newly 54 weeks cumul	2347.	16267.3	{8507., 22551.}	593.112	0.783938
39	newly 3 weeks spec	1569.	3795.13	{1884., 6699.}	142.392	0.339112	newly 3 weeks cumul	6961.	14403.3	{7332., 24446.}	108.655	0.283755	newly 5 weeks spec	719.	2356.13	{1056., 4191.}	227.695	0.451412	newly 5 weeks cumul	8738.	19751.6	{10192., 34092.}	126.807	0.315519	newly 48 weeks cumul	10021.	27612.	{13308., 47325.}	175.621	0.386282
40	newly 3 weeks spec	2069.	2322.17	{1094., 4123.}	54.3064	0.204811	newly 3 weeks cumul	8421.	9526.14	{4728., 17010.}	50.4782	0.190308	newly 5 weeks spec	1045.	1263.58	{588., 2244.}	59.9279	0.208203	newly 5 weeks cumul	10908.	12507.	{6067., 22262.}	52.3369	0.193897	newly 49 weeks cumul	13560.	15746.7	{7549., 27996.}	54.1722	0.197407
41	newly 3 weeks spec	1243.	2770.01	{1278., 5891.}	123.959	0.282124	newly 3 weeks cumul	5945.	11421.	{5774., 23400.}	94.7821	0.239601	newly 5 weeks spec	608.	1454.41	{638., 3122.}	140.756	0.297933	newly 5 weeks cumul	7464.	14895.5	{7333., 30833.}	101.861	0.24872	newly 50 weeks cumul	8541.	18377.	{8898., 38360.}	116.601	0.271688
42	newly 3 weeks spec	457.	997.179	{503., 1425.}	118.854	0.299433	newly 3 weeks cumul	2671.	5101.39	{2704., 7414.}	92.686	0.248194	newly 5 weeks spec	82.	384.91	{206., 537.}	369.403	0.627117	newly 5 weeks cumul	2987.	6108.96	{3205., 8867.}	105.434	0.273678	newly 54 weeks cumul	3092.	6745.85	{3561., 9810.}	118.674	0.299611
43	newly 3 weeks spec	2726.	4173.14	{2516., 7253.}	57.5897	0.17479	newly 3 weeks cumul	11008.	15509.1	{9848., 25253.}	46.1562	0.150414	newly 5 weeks spec	1377.	2605.48	{1497., 4651.}	90.6186	0.242503	newly 5 weeks cumul	14359.	21444.1	{13274., 35943.}	53.5912	0.166933	newly 47 weeks cumul	18000.	30024.2	{17972., 51943.}	69.1006	0.200446
44	newly 3 weeks spec	1026.	1067.45	{556., 1942.}	41.6711	0.173701	newly 3 weeks cumul	5335.	5231.95	{2731., 9299.}	39.9281	0.176731	newly 5 weeks spec	393.	442.561	{226., 841.}	45.2733	0.173351	newly 5 weeks cumul	6350.	6361.42	{3330., 11386.}	40.4715	0.175327	newly 53 weeks cumul	7041.	7151.62	{3720., 12885.}	40.8097	0.174346
45	newly 3 weeks spec	374.	2835.03	{1365., 4835.}	658.029	0.813268	newly 3 weeks cumul	2546.	12434.5	{6447., 21056.}	388.394	0.634954	newly 5 weeks spec	135.	1329.44	{631., 2271.}	884.769	0.917377	newly 5 weeks cumul	2913.	15709.9	{7960., 26662.}	439.302	0.674062	newly 52 weeks cumul	3058.	18415.7	{9188., 31234.}	502.213	0.718738
46	newly 3 weeks spec	1322.	4025.64	{2124., 6830.}	204.511	0.427851	newly 3 weeks cumul	7028.	16122.	{9067., 25940.}	129.396	0.326437	newly 5 weeks spec	520.	2194.62	{1014., 3486.}	322.043	0.538696	newly 5 weeks cumul	8416.	21308.3	{11478., 36081.}	153.188	0.360679	newly 52 weeks cumul	9218.	26522.1	{13568., 44405.}	187.72	0.405902
47	newly 3 weeks spec	877.	2401.03	{1303., 3971.}	173.803	0.383744	newly 3 weeks cumul	5116.	11394.2	{6392., 18858.}	123.169	0.302977	newly 5 weeks spec	280.	1023.78	{520., 1743.}	265.636	0.502514	newly 5 weeks cumul	5861.	13996.7	{7704., 23117.}	139.124	0.330536	newly 54 weeks cumul	6385.	15829.2	{8608., 26188.}	148.134	0.344845
48	newly 3 weeks spec	1336.	2825.19	{1418., 4666.}	113.464	0.260606	newly 3 weeks cumul	7014.	12747.2	{6947., 21527.}	84.6052	0.22212	newly 5 weeks spec	498.	1242.17	{598., 2051.}	151.461	0.3126	newly 5 weeks cumul	8264.	15889.4	{8459., 26749.}	94.7166	0.237856	newly 54 weeks cumul	8948.	18148.9	{9483., 30300.}	104.852	0.254329
49	newly 3 weeks spec	215.	899.726	{491., 1639.}	318.477	0.574875	newly 3 weeks cumul	1583.	5367.07	{2891., 9500.}	239.044	0.483782	newly 5 weeks spec	43.	273.603	{152., 477.}	536.285	0.755551	newly 5 weeks cumul	1735.	6141.95	{3331., 10856.}	254.003	0.502585	newly 56 weeks cumul	1768.	6479.79	{3494., 11435.}	266.504	0.517552
50	newly 3 weeks spec	2185.	3062.09	{1563., 5356.}	54.7783	0.166534	newly 3 weeks cumul	9176.	11854.8	{6203., 20249.}	45.9742	0.152489	newly 5 weeks spec	1236.	1799.59	{887., 3217.}	60.9754	0.177192	newly 5 weeks cumul	12158.	16005.3	{8245., 27620.}	48.5469	0.157062	newly 47 weeks cumul	15907.	21483.9	{10874., 37432.}	51.9089	0.162679
